# Chronic hypersensitivity pneumonitis is associated with an increased risk of venous thromboembolism: a retrospective cohort study

**DOI:** 10.1186/s12890-021-01794-y

**Published:** 2021-12-17

**Authors:** Małgorzata Sobiecka, Monika Szturmowicz, Katarzyna Lewandowska, Agata Kowalik, Ewa Łyżwa, Katarzyna Zimna, Inga Barańska, Lilia Jakubowska, Jan Kuś, Renata Langfort, Witold Tomkowski

**Affiliations:** 1 1st Department of Lung Diseases, National Tuberculosis and Lung Diseases Research Institute, Plocka 26, 01-138 Warsaw, Poland; 2 Department of Radiology, National Tuberculosis and Lung Diseases Research Institute, Warsaw, Poland; 3 Department of Pathology, National Tuberculosis and Lung Diseases Research Institute, Warsaw, Poland

**Keywords:** Idiopathic pulmonary fibrosis, Chronic hypersensitivity pneumonitis, Venous thromboembolism, Deep vein thrombosis, Pulmonary embolism

## Abstract

**Background:**

Idiopathic pulmonary fibrosis (IPF) and chronic hypersensitivity pneumonitis share commonalities in pathogenesis shifting haemostasis balance towards the procoagulant and antifibrinolytic activity. Several studies have suggested an increased risk of venous thromboembolism in IPF. The association between venous thromboembolism and chronic hypersensitivity pneumonitis has not been studied yet.

**Methods:**

A retrospective cohort study of IPF and chronic hypersensitivity pneumonitis patients diagnosed in single tertiary referral center between 2005 and 2018 was conducted. The incidence of symptomatic venous thromboembolism was evaluated. Risk factors for venous thromboembolism and survival among those with and without venous thromboembolism were assessed.

**Results:**

A total of 411 (259 IPF and 152 chronic hypersensitivity) patients were included (mean age 66.7 ± 8.4 vs 51.0 ± 13.3 years, respectively). There were 12 (4.6%) incident cases of venous thromboembolism in IPF and 5 (3.3%) in chronic hypersensitivity pneumonitis cohort. The relative risk (RR) of venous thromboembolism in chronic hypersensitivity pneumonitis was not significantly different to that found in patients with IPF (7.1 vs 11.8/1000 person-years, RR 1.661 95% CI 0.545–6.019, respectively).

The treatment with systemic steroids (OR 5.38; 95% CI 1.65–18.8, *p* = 0.006) and GAP stage 3 (OR 7.85; 95% CI 1.49–34.9; *p* = 0.037) were significant risk factors for venous thromboembolism in IPF. Arterial hypertension and pulmonary hypertension significantly increased risk of venous thromboembolism in chronic hypersensitivity pneumonitis. There were no significant differences in survival between patients with and without venous thromboembolism.

**Conclusions:**

The patients with chronic hypersensitivity pneumonitis have a marked increase in the risk of venous thromboembolism, similar to the patients with IPF. Venous thromboembolism does not affect the survival of patients with IPF and chronic hypersensitivity pneumonitis.

**Supplementary Information:**

The online version contains supplementary material available at 10.1186/s12890-021-01794-y.

## Background

Chronic hypersensitivity pneumonitis (cHP), is a complex diffuse parenchymal disease caused by repeated and prolonged exposure to inhaled antigens in susceptible individuals, leading to an inflammatory response in the lungs, and, in some patients, to development of pulmonary fibrosis [1]. The experimental study on bronchoalveolar lavage fluid (BALF) of patients with hypersensitivity pneumonitis has demonstrated that the alveolar haemostatic balance is shifted in favour of procoagulant and antifibrinolytic activity [2], similarly to patients with idiopathic pulmonary fibrosis (IPF), the most common progressive fibrotic disorder of unknown aetiology and unfavourable prognosis [3, 4]. Extensive research over the last two decades have contributed to a better understanding of the pathogenesis of this rare, debilitating disease. In genetically susceptible ageing individuals, the repeated micro injury of the alveolar epithelium has been recognized as the first driver of an altered, aberrant repair process, initiating a complex cascade of events leading to scarring of lung tissue. In the early phase of wound healing process, epithelial and endothelial cells damage result in the activation of coagulation cascade via the tissue factor (TF)-dependent extrinsic pathway. As a result, the balance between coagulation and fibrinolysis is shifted in favour of procoagulant activity, enhanced by increased levels of fibrinolysis inhibitors as plasminogen activator inhibitor 1 and 2 (PAI1 and PAI2), and protein-C inhibitors [5, 6]. An imbalance between thrombosis and fibrinolysis has been demonstrated in animal models of lung fibrosis and in the alveolar compartment of IPF patients [7, 8]. Furthermore, several population-based studies of the last decade showed an association between IPF and venous thromboembolism (VTE), indicating an increased risk of prothrombotic state, increased incidence and prevalence of VTE in IPF patients, and ultimately increased mortality [9–13].

The association between chronic hypersensitivity pneumonitis and venous thromboembolism has not been studied yet. The aim of this study was to evaluate the epidemiology of VTE in IPF and cHP patients, diagnosed in our tertiary referral centre and further to evaluate related risk factors. We also examined overall survival of patients with VTE compared to non-VTE group.

## Methods

### Statement of ethics

The study was approved by the Bioethical Committee of the National Tuberculosis and Lung Diseases Research Institute in Warsaw, Poland (approval No. KB-36 /2019) and conducted in accordance with the Declaration of Helsinki.

### Patients

**Fig. 1:**
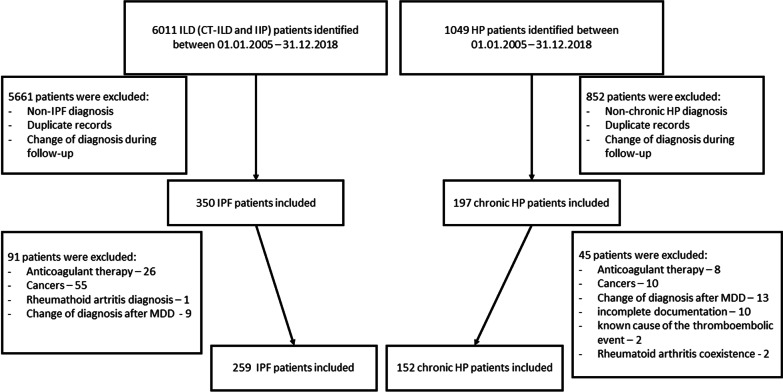
Flowchart of the study population selection. ILD, interstitial lung disease; CTD-ILD, connective tissue disease-associated interstitial lung disease; IIP, idiopathic interstitial pneumonia; HP, hypersensitivity pneumonitis; IPF, idiopathic pulmonary fibrosis; MDD, multidisciplinary discussion

A retrospective analysis of an interstitial lung disease (ILD) database of the single pulmonary department identified 350 consecutive patients diagnosed with IPF and 197 patients diagnosed with HP, during the period of January 1, 2005, to December 31, 2018. Flowchart illustrating the selection of patients to the final study population is shown on Fig. 1.

The patients with IPF were re-evaluated in a multidisciplinary team discussion if they would fulfil the currently published guidelines [14]. The diagnosis of HP was established based on combination of clinical, radiological and histopathological features and laboratory findings. The applied diagnostic criteria of HP recognition were as follows: 1. Identification of occupational or environmental exposure to possible antigens responsible for the symptoms of the disease (detailed anamnesis) and /or the presence of specific IgG antibodies against various antigens in serum of symptomatic patients, 2. Characteristic pattern of radiological changes on chest CT, 3. Increased percentage of lymphocytes exceeding 30% in bronchoalveolar fluid and 4. Histopathology suggestive of HP in the specimen obtained during transbronchial or surgical lung biopsy (if the criteria 1 + 2 + 3 were not fulfilled) [15–17].

Only patients with chronic HP diagnosed according to criteria of Vasakova et al. were enrolled into the study [17]. The detailed description of diagnostic procedures used by our group has been published previously [18]. Moreover, patients receiving anticoagulation treatment as well as those with a history of VTE or known risk factors for VTE (acquired or inherited thrombophilia, recent surgery, major trauma, limb fracture, malignancy, connective tissue diseases, vasculitis, nephrotic syndrome, pregnancy) were excluded.

The plasma D-dimer concentration and all assessed medical parameters were obtained as routine measurements in patients with ILDs during the diagnostic process.

### Study endpoints

The primary outcomes were symptomatic deep vein thrombosis (DVT), and/or pulmonary embolism (PE) documented in the medical records after the diagnosis of IPF or cHP had been made. DVT was defined as the presence of thrombus of the femoral or popliteal veins or distal thrombosis (calf) with proximal extension on Doppler ultrasound. PE was diagnosed as the presence of embolus on a thorax computed tomography (CT) angiogram.

The secondary outcome measures were the factors predisposing to VTE in patients with IPF or cHP and the impact of VTE on all-cause mortality.

The vital status of all patients was verified in November 2019 by review of subsequent patients visits or by the data on deaths obtained from the Ministry of Digital Affairs. Survival time was calculated from diagnosis to death or to the end of observation time.

### Statistical analysis

Basic characteristics of cHP and IPF groups were compared using chi-square and *t*-Student tests. Incidence of VTE in IPF and cHP groups was expressed as a number per 1000 person-years. Differences between groups were expressed as relative risk (RR) with respective 95% confidence interval (95% CI). Potential risk factors of VTE event among IPF and cHP patients were described using odds ratios and its 95% confidence intervals. Overall survival of patients in cHP/IPF and in VTE/non-VTE groups was described using Kaplan–Meier estimates and expressed as median, 3-year and 5-year survival with respective 95% confidence intervals. Significance of between-groups differences was assessed using log-rank test. In all analyses value of 0.05 was set up as a significance level. All computations were performed using R version 4.0.2 statistical software [19].

## Results

### Baseline characteristics of patients

The study population ultimately consisted of 411 patients: 259 cases with a multidisciplinary diagnosis of IPF and 152 cases with the cHP diagnosis (Fig. 1). The demographic and baseline characteristics of these patients are shown in Table 1 and the detailed characteristics of cHP patients are presented in Additional file 1: Table S1.

**Table 1:** Demographics and clinical characteristics of patients with idiopathic pulmonary fibrosis and chronic hypersensitivity pneumonitis at diagnosis

Characteristics	IPF (n = 259)	cHP (n = 152)	*P*
Age, years	66.7 (8.4)	51.0 (13.3)	< 0.001
Male gender	181 (69.9)	76 (50.0)	< 0.001
*Smoking status*			
Non-smoker	78 (30.1)	103 (67.8)	< 0.001
Current or former smoker	181 (69.9)	49 (32.2)	
BMI, kg/m^2^	28.3 (3.8)	27.3 (4.9)	0.024
*Comorbidities*			
Arterial hypertension	159 (61.4)	91 (59.9)	0.834
Coronary artery disease	79 (30.5)	14 (9.2)	< 0.001
Diabetes mellitus	62 (23.9)	23 (15.1)	0.043
Congestive heart failure	47 (18.1)	15 (9.9)	0.032
Atrial fibrillation	10 (3.9)	1 (0.7)	0.061
Pulmonary hypertension (by echocardiography)	97 (48.5)	44 (33.6)	0.009
GERD	43 (16.6)	12 (7.9)	0.016
Emphysema	77 (29.8)	23 (16.2)	0.003
Stroke	7 (2.7)	3 (2.0)	0.751
OSA	6 (2.3)	3 (2.0)	1.000
*Treatment*			
ASA	88 (34.1)	19 (12.6)	< 0.001
Systemic steroid	58 (22.4)	137 (90.1)	< 0.001
Immunosuppressants	34 (13.1)	51 (33.6)	< 0.001
*Pulmonary function test, % predicted*			
FVC	87.3 (20.0)	80.9 (22.2)	0.003
FEV_1_	89.5 (20.4)	75.8 (20.7)	< 0.001
TLC	81.9 (16.6)	86.7 (22.2)	0.013
DLco	52.6 (16.8)	51.2 (18.4)	0.430
6-min walking distance (m)	424.5 (123.0)	481.6 (114.4)	< 0.001
D-dimer (µg/ml)	935.5 (2078.6)	516.6 (574.2)	0.027
TVPG mmHg	35.1 (12.5)	30.2 (9.7)	< 0.001
CT angiogram performed	64 (24.7)	37 (24.3)	1.000
Follow-up time, years	3.9 (3.3)	4.6 (3.5)	0.042

Data are expressed as n (%) or mean (SD) unless otherwise specified

ASA, acetylsalicylic acid; BMI, body mass index; CT, computed tomography; FVC, forced vital capacity; FEV_1_, forced expiratory volume in one second; TLC, total lung capacity; DLco, diffusing capacity of the lung for carbon monoxide; GERD, gastroesophageal reflux disease; OSA, obstructive sleep apnoea; TVPG, tricuspid valvular pressure gradient; IPF, idiopathic pulmonary fibrosis; cHP, chronic hypersensitivity pneumonitis

The patients with IPF were older, more often men, smokers, and had more comorbidities comparing to patients with cHP. There were also significant differences in terms of the treatment with glucocorticoids, immunosuppressants and acetylsalicylic acid (ASA), results of pulmonary function tests, distance covered in the 6-min walk test, the plasma D-dimer concentration and tricuspid valve regurgitation pressure gradient (TVPG) assessed in echocardiography between these two groups. Antifibrotics (pirfenidone and nintedanib) were used only in the IPF patients for a relatively short period of time: 41 patients were treated with pirfenidone and 21 cases with nintedanib.

CT angiography was performed in comparable percentage of patients in the both groups (IPF- 24.7 vs 24.3% in cHP group, *p* = 1.000). The mean follow-up period was significantly shorter in people with IPF as compared to individuals with cHP (mean (SD): 3.9 (3.3) vs 4.6 (3.5) years, *p* = 0.042).

### Incidence of venous thromboembolism

We identified 12 (4.6%) symptomatic events of VTE in IPF patients. Seven (2.7%) patients were diagnosed with DVT of the lower extremity and 8 (3.1%) patients were diagnosed with PE. Of these, 3 (1.2%) patients had both DVT and PE. In turn, five (3.3%) patients developed VTE event after the cHP diagnosis. There were 3 (2.0%) patients with DVT, 4 (2.6%) with PE, and 2 (1.3%) with both DVT and PE in this group. No patient had recurrent VTE event. The patients in VTE group were treated with various anticoagulants (low molecular weight heparin, new oral anticoagulants, Vitamin K antagonists). During follow-up only 1 patient in VTE group had a non-major bleeding—small epistaxis. The detailed characteristics of all patients with venous thromboembolic event were presented in Additional file 1: Table S2.

After recalculation of the incidence of new symptomatic thromboembolic events in the IPF and cHP group per 1000 person-years of follow-up, it was shown that the relative risk of a thromboembolic event in the cHP group was similar to that in patients with IPF (Table 2).

**Table 2:** Comparison of the incidence of venous thromboembolism in IPF and cHP groups per 1000 person-years of follow-up

	Person-years	Pulmonary embolism		Deep vein thrombosis		Venous thromboembolism	
	N	N	N/1000 person-years	N	N/1000 person-years	N	N/1000 person-years
IPF	1020	8	7.8	7	6.9	12	11.8
cHP	706	4	5.7	3	4.2	5	7.1
RR (95% CI)		1.384 (0.371–6.282) *p* = 0.83*		1.615 (0.369–9.679) *p* = 0.72*		1.661 (0.545–6.019) *p* = 0.48*	

RR, relative risk; CI, confidence interval; IPF, idiopathic pulmonary fibrosis; cHP, chronic hypersensitivity pneumonitis

*Exact Fisher test

### Risk factors of venous thromboembolism in IPF and cHP group

The risk factors of VTE were evaluated separately in IPF and cHP group using univariate logistic regression model. Our analysis revealed that the treatment with systemic steroids (OR 5.38; 95% CI 1.65–18.8, *p*=0.006) and GAP stage III (OR 7.85; 95% CI 1.49–34.9; *p*=0.037) were significant risk factors for VTE in patients with IPF (Table 3). In cHP group arterial hypertension (OR>100, CI 0.00 to NA; *p*=0.022) and pulmonary hypertension (OR 8.6; 95% CI 1.22–171, *p*=0.030) significantly increased the risk of VTE (Table 4). On the other hand, the higher FEV_1_% FVC index significantly reduced the risk of VTE (OR 0.92; 95% CI 0.87–0.98; *p* = 0.008) in patients with cHP.

**Table 3:** Predictors of venous thromboembolism in IPF group

Characteristic	Non-VTE	VTE	OR	95% CI	*p*-value
No. of patients	247 (95.4)	12 (4.6)			
Age, median (IQR) years	67 (61–72)	72 (62–74)	1.03	0.96–1.11	0.408
Male gender	174 (70)	7 (58)	0.59	0.18–2.04	0.385
*Smoking*					
Smokers or ex-smokers	174 (70)	7 (58)	0.59	0.18–2.04	0.385
*Comorbidities*					
Arterial hypertension Coronary artery disease Diabetes mellitus Congestive heart failure Pulmonary hypertension (by echocardiography)	146 (60) 73 (30) 59 (24) 44 (18) 89 (47)	10 (83) 6 (50) 3 (25) 3 (25) 8 (67)	3.29 2.38 1.06 1.54 2.22	0.84–21.7 0.72–7.86 0.23–3.69 0.33–5.40 0.68–8.57	0.090 0.149 0.930 0.545 0.191
*Treatment*					
ASA Systemic steroids Immunosuppressants	85 (35) 51 (21) 30 (12)	3 (25) 7 (58) 4 (33)	0.63 5.38 3.62	0.14–2.18 1.65–18.8 0.92–12.2	0.485 0.006 0.064
*Pulmonary function test*					
FVC% predicted FEV_1_% predicted FEV_1_%FVC DLco% predicted	87 (74–102) 89 (77–103) 81 (76–75) 51 (40–65)	78 (70–92) 82 (71–94) 79 (78–86) 41 (35–47)	0.98 0.98 1.01 0.97	0.94–1.01 0.95–1.01 0.94–1.09 0.92–1.01	0.128 0.211 0.759 0.104
6-min walking distance (m)	430 (352–511)	331 (270–401)	0.99	0.99–1.00	0.061
*GAP stage*					
I II III	157 (64) 77 (32) 10 (4)	6 (55) 2 (18) 3 (27)	– 0.68 7.85	– 0.10–3.03 1.49–34.9	0.037
D-dimer (µg/ml)	483 (325–731)	1095 (478–1779)	1.02	1.00–1.03	0.111

Statistics presented: n (%) or median (IQR)

OR, odds ratio; CI, confidence interval; ASA, acetylsalicylic acid; BMI, body mass index; GAP, gender-age-physiology; FVC, forced vital capacity; FEV_1_, forced expiratory volume in one second; DLco, diffusing capacity of the lung for carbon monoxide; IPF, idiopathic pulmonary fibrosis; VTE, venous thromboembolism

**Table 4:** Predictors of venous thromboembolism in cHP group

Characteristic	Non-VTE	VTE	OR	95% CI	*p*-value
No. of patients	147 (96.7)	5 (3.3)			
Age; median (IQR); years	52 (43–61)	53 (52–54)	1.02	0.95–1.10	0.653
Male gender	72 (49)	4 (80)	4.17	0.60–82.6	0.128
*Smoking*					
Smokers or ex-smokers	46 (31)	3 (60)	3.29	0.53–25.6	0.195
*Comorbidities*					
Arterial hypertension Coronary artery disease Diabetes mellitus Congestive heart failure Pulmonary hypertension (by echocardiography)	86 (59) 14 (9.5) 23 (16) 14 (9.5) 40 (32)	5 (100) 0 (0) 0 (0) 1 (20) 4 (80)	> 100 0.00 0.00 2.37 8.60	0.00–NA – – 0.12–17.5 1.22–171	0.022 0.321 0.196 0.489 0.030
*Treatment*					
ASA Systemic steroids Immunosuppressants	19 (13) 132 (90) 49 (33)	0 (0) 5 (100) 2 (40)	0.00 > 100 1.33	– 0.00–NA 0.17–8.30	0.242 0.304 0.759
*Pulmonary function test*					
FVC% predicted FEV_1_% predicted FEV_1_%FVC DLco% predicted	80 (64–98) 77 (61–91) 82 (76–86) 49 (40–62)	77 (67–90) 66 (50–72) 70 (48–77) 58 (32–67)	0.99 0.97 0.92 1.00	0.95–1.03 0.93–1.02 0.87–0.98 0.95–1.05	0.751 0.234 0.008 0.937
6-min walking distance (m)	487 (430–568)	413 (310–432)	0.99	0.99–1.00	0.163
D-dimer (µg/ml)	392 (242–535)	255 (182–379)	0.84	0.48–1.08	0.314

Statistics presented: n (%) or median (IQR)

OR, odds ratio; CI, confidence interval; ASA, acetylsalicylic acid; BMI, body mass index; FVC, forced vital capacity; FEV_1_, forced expiratory volume in one second; DLco, diffusing capacity of the lung for carbon monoxide; VTE, venous thromboembolism; cHP, chronic hypersensitivity pneumonitis

### Survival analysis

The median survival time in IPF patients was significantly shorter in comparison to cHP patients (79.9 vs 121.5 months, respectively, *p* < 0.001) (Table 5).

**Table 5:** Survival analysis of patients depending on the underlying disease and thromboembolic events

Variable	Group	No of patients	No of deaths	Median survival (95% CI)	3-year survival (95% CI)	5-year survival (95% CI)	*p*-value log-rank test
Disease	cHP	152	38	121.5 (110.1–NA)	96.16 (92.91–99.54)	90.05 (84.16–96.35)	< 0.001
	IPF	259	116	79.9 (68.7–105.8)	81.36 (76.33–86.73)	60.78 (53.94–68.47)	
VTE	No	394	146	105.8 (90.6–117.9)	86.61 (82.97–90.41)	71.86 (66.56–77.57)	0.593
	Yes	17	8	60.7 (46.5–NA)	87.84 (73.37–100.00)	55.29 (33.46–91.34)	

CI, confidence interval; cHP, chronic hypersensitivity pneumonitis; IPF, idiopathic pulmonary fibrosis; VTE, venous thromboembolism

**Fig. 2:**
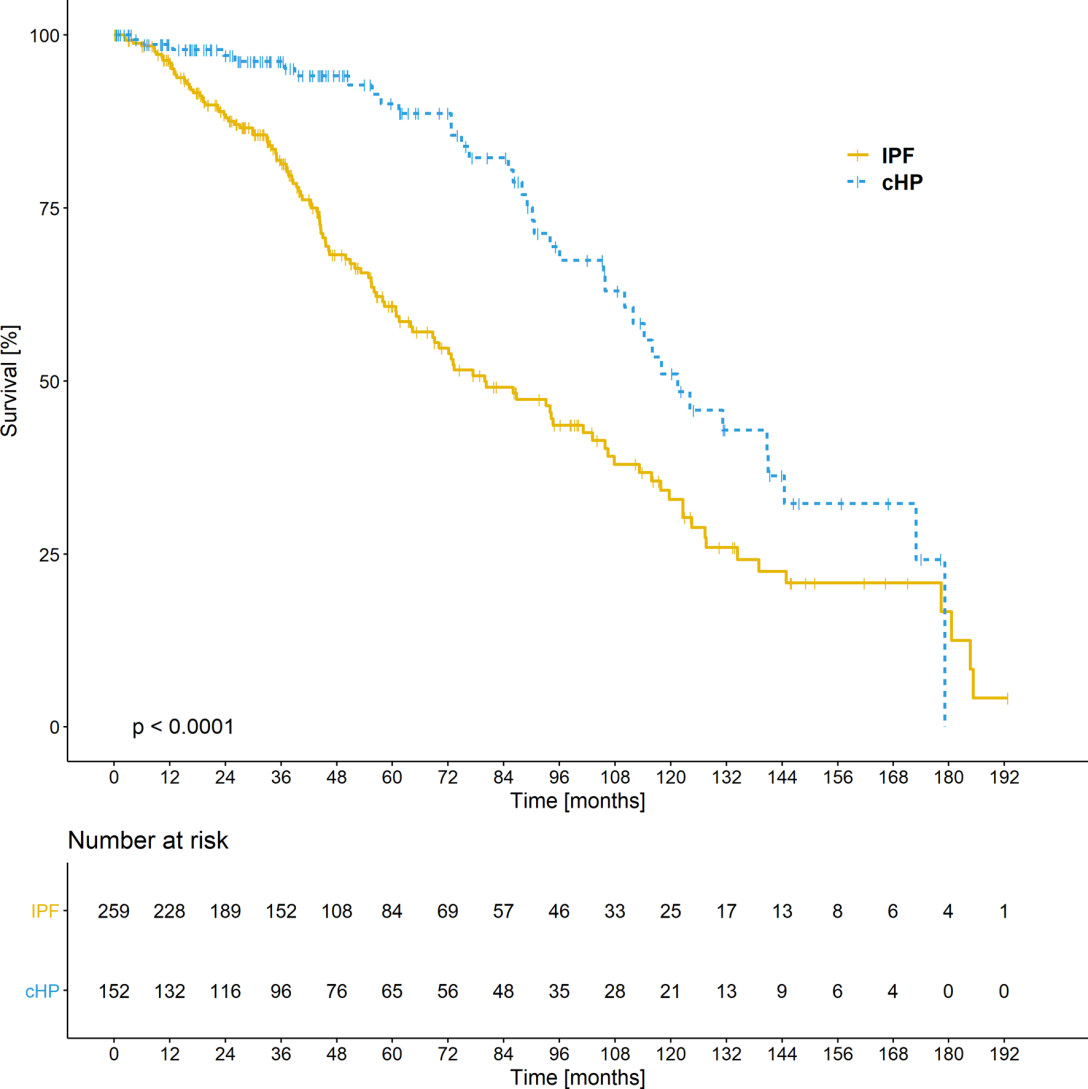
Kaplan–Meier curves for overall survival. Using the log-rank test as univariate analysis there was a significant difference between the patients with idiopathic pulmonary fibrosis (IPF) and chronic hypersensitivity pneumonitis (cHP)

**Fig. 3:**
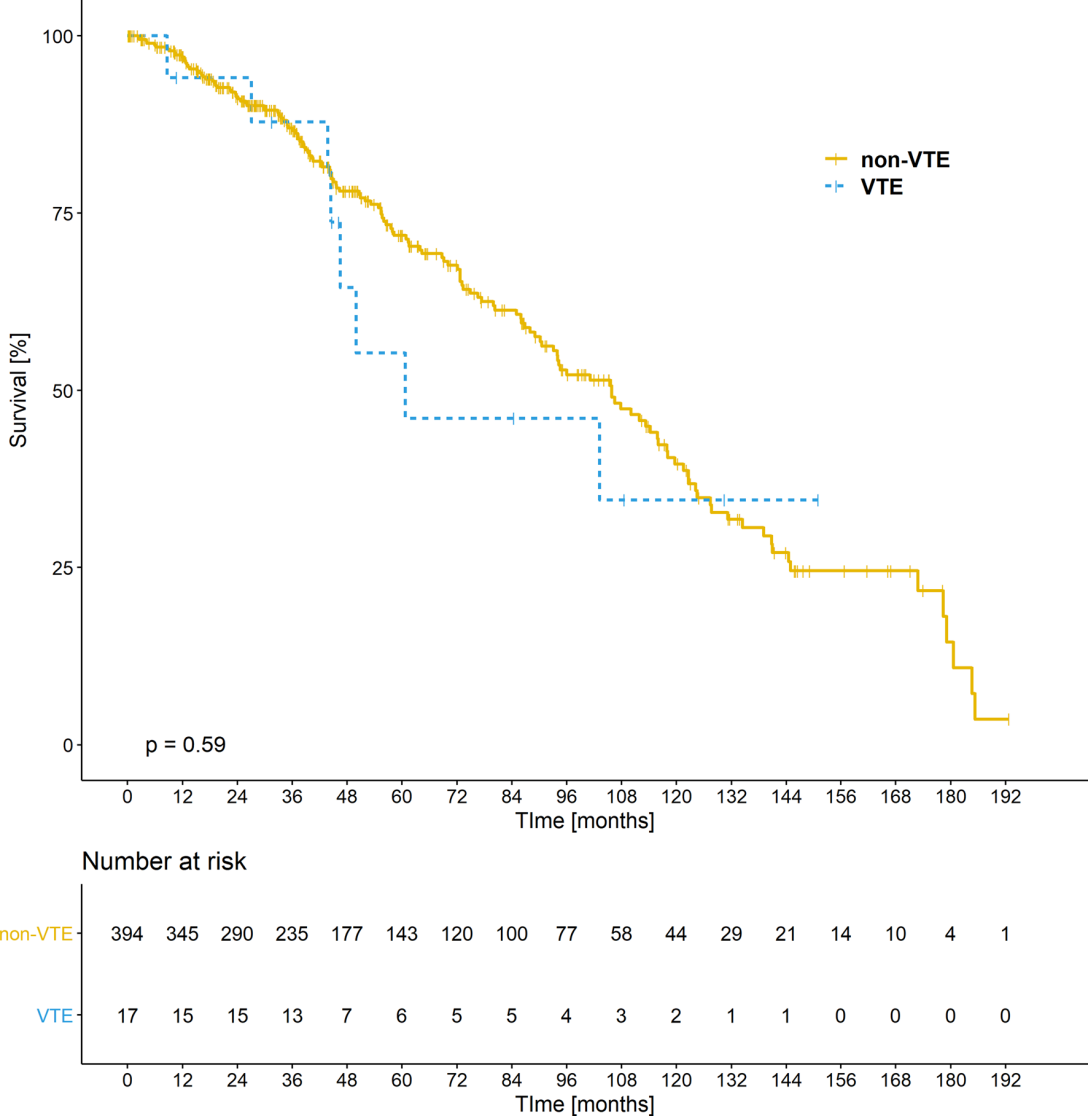
Kaplan–Meier survival curves of patients with idiopathic pulmonary fibrosis (IPF) and chronic hypersensitivity pneumonitis (cHP), with and without venous thromboembolism (VTE) (log-rank test, *p* = 0.59)

During the follow-up period, 116 of the 259 (45%) individuals with IPF and 38 of the 152 (25%) people with cHP died. The Kaplan–Meier curves in Fig. 2 show survival amongst individuals with IPF and cHP. The probability of 3- and 5-years survival, and the median survival did not significantly differ between the patients with and without VTE (Table 5). The Kaplan–Meier curves for overall survival amongst the patients with and without VTE event are shown on Fig. 3.

## Discussion

We found that the incidence rate of symptomatic venous thromboembolism in patients with chronic hypersensitivity pneumonitis was similar to that demonstrated in IPF group (7.1 per 1000 person-years vs 11.8 per 1000 person-years, RR 1.661 95% CI 0.545-6.019, *p*=0.48, respectively), even though the patients with IPF were older and had more comorbidities. We also showed that there were no significant differences in median, 3- and 5-years survival rate between the patients with and without VTE. To our knowledge, this is the first large comprehensive study on epidemiology of venous thromboembolism performed in well characterized cohort of cHP and IPF.

Venous thromboembolism, comprising deep vein thrombosis and pulmonary embolism, is a common, preventable disease with a substantial morbidity and mortality. An estimated incidence rate of VTE in the general population range from 1.04 to 2.7 per 1000 person-years [20–25]. In the present study we showed that the incidence rate of symptomatic VTE events in patients with cHP was similar to that demonstrated in IPF group and was higher than the rate found in aforementioned population-based studies. Additionally, our results regarding the IPF group are similar to those presented in other studies indicating an increased risk and incidence of PE and DVT in patients with IPF. In large population-based study, Dalleywater et al. [13] found that people with IPF had higher incidence rates of PE and DVT, compared to the general population (PE 9.3 vs 1.52/1000 person-years, and DVT 4.3 vs 2.11/1000 person-years, *p*<0.001, respectively). A similar, large increase in the incidence of DVT (5.9/1000 person-years) was demonstrated in patients with IPF by Hubbard et al. [9]. We found comparable incidence rates of PE and DVT in both evaluated cohorts IPF and cHP (PE: 7.8 vs 5.7/1000 person-years, RR 1.384 95% CI 0.371-6.282, and DVT 6.9 vs 4.2/1000 person-years, RR 1.615, 95% CI 0.369-9.679, respectively), thought the patients with cHP were younger, had lower BMI and less comorbidities. In recent years, a growing body of evidence indicate that the cHP and IPF may share commonalities in the mechanism involved in their pathogenesis and progression. Pathogenetic mechanism of lung fibrosis seen in IPF and cHP is triggered by repetitive lung parenchyma injuries caused in genetically susceptible individuals by the environmental factors [26]. Additionally, some genetic variants such as shortened telomeres and the MUC5B promoter variant rs35705950 that contribute to IPF susceptibility, have been linked to cHP as well [27]. Tissue injury initiates a cascade of events, one of the earliest of which is activation of the clotting system [5]. There is good evidence that the coagulation cascade is activated in several fibrotic lung diseases, regardless of the factor initiating lung fibrosis, including IPF [7, 8] and HP [2].

Nevertheless, the increased risk of VTE in cHP cannot be explained only by the similarity of both diseases and it is also possible that elements unique to the disease may play a role. HP typically results from an immune-mediated reaction induced by an inhaled antigen leading to predominantly lymphocytic inflammatory pattern with granulomatous inflammation. However, neutrophilic inflammation may play a role early in the disease course and during subsequent fibrosis [1]. Chronic inflammation, as a weak risk factor for venous thromboembolism, is known to provoke a hypercoagulable state similar to fibrosis. Activated leukocytes are the primary source of procoagulant tissue factor-positive microparticles that might locally activate the coagulation cascade. Moreover, neutrophils produce neutrophil extracellular traps (NETs) composed of DNA, histones, and microbial proteins that promote thrombus formation by providing a scaffold for red blood cells, platelets, and procoagulant molecules [28].

The relationship between IPF and VTE or prothrombotic state was the subject of several epidemiological studies in the past [9–13]. In large, general population-based study of people with IPF, Hubbard et al. [9] found an increased risk of having DVT in patients with IPF compared with the general population both before and after the diagnosis of interstitial lung disease was first recorded. Subsequent studies in Denmark and the United States have confirmed these results. Sode et al. [10] examined the entire Danish population from 1980 through 2007, using national registries to identify patients with interstitial pneumonia and VTE events. They showed that prior history of VTE was associated with an increased risk of developing idiopathic interstitial pneumonia, especially among those never treated with anticoagulants. An elevated risk of thromboembolic disease in patients with IPF was also demonstrated in the study using mortality data from all USA decedents [11]. The authors identified IPF cases and three groups for comparison purposes: population based-controls, chronic obstructive pulmonary disease (COPD) patients and lung cancer patients. They found that decedents with IPF had a significantly greater risk of VTE than the comparison groups and that those with VTE and pulmonary fibrosis died at a younger age than those with pulmonary fibrosis alone. Moreover, a meta-analysis of 5 studies on the relationship between VTE and IPF showed more than a twofold increase in the risk of VTE in patients with IPF [29].

Hitherto, the association between VTE and cHP has not been studied yet. After a careful search of the medical databases as PubMed, MEDLINE and Web of Science, we found only one case report regarding the association of acute HP due to Aspergillus hypersensitization and recurrent pulmonary embolism [30]. The authors hypothesized that the inflammation induced by bioaerosol exposure causing HP was a temporal trigger to venous thrombotic events in their patient. Additionally, the research paper from the tertiary referral centre for ILDs has recently been published concerning the association between various comorbid conditions and survival in cHP [31]. The authors have found that thromboembolic disorders occurred in 4.7% of patients with cHP and were associated with an improved survival. In our cHP group VTE was identified in 3.3% of cases. In contrary to the study of Wälscher et al., where the prevalence of thromboembolic disorders was assessed, we evaluated the incidence rate of VTE events in our cohorts, and additionally the patients with the known risk factors for thromboembolism as well as being treated with anticoagulation were excluded from the analysis.

The results of our study support the previous evidence of increased risk of VTE in patients with IPF, and additionally provide new data on the increased risk of DVT and PE with cHP, comparable to that of demonstrated in IPF. The possible explanation of these relationships might be, apart from the similar pathogenetic mechanisms resulting in the shift of the haemostasis balance in favour of the procoagulant and antifibrinolytic activity, simply the reduced mobility of patients with IPF and cHP due to respiratory symptoms and reduced exercise capacity. Furthermore, the increased risk of VTE could result from the exposure to glucocorticoids, the medication often applied in the treatment of cHP and in the past used in patients with IPF. The approach to IPF treatment changed in 2012 after the publication of the results of the PANTHER-IPF clinical trial, in which participants treated with the combined three-drug regimen of prednisone, azathioprine, and *N-*acetylcysteine, compared to the placebo had higher mortality, more hospitalizations and serious adverse events [32]. As our retrospective analysis included patients with IPF diagnosed between 2005 and 2012, some of them were treated with glucocorticosteroids and azathioprine. Our analysis revealed that the treatment with systemic steroids and more advanced disease were significant risk factors for VTE in patients with IPF. This was not demonstrated in the cHP group, where the vast majority of patients were treated with systemic steroids. On the other hand, it is worth mentioning that all patients with confirmed VTE in the cHP group were treated with systemic steroids. The association between glucocorticoids and VTE was examined in a nationwide population-based case-control study in the past. The authors found that glucocorticoid users had a dose-dependent increased risk of VTE [33].

We found that in cHP group the presence of arterial hypertension and pulmonary hypertension significantly increased risk of VTE. In the study evaluating the epidemiology of VTE in large, well-characterized cohort of patients with systemic sclerosis, Johnson et al. [34] reported that the presence of pulmonary arterial hypertension (PAH) was an independent risk factor for VTE and predictor of mortality in this disorder. Similar as in their study, this risk factor (and the other baseline characteristics we evaluated) was present prior to the occurrence of VTE. This may be of importance in the evaluation of patients with cHP who show the echocardiographic signs of pulmonary hypertension and have symptoms of worsening dyspnoea, where the clinicians should maintain a high index of suspicion for VTE.

Finally, we assessed the impact of VTE on survival concomitantly in the IPF and cHP groups due to the low number of VTE cases. We did not find any significant differences in median, 3- and 5-years survival between those with and without VTE. The exclusion of known risk factors for VTE, such as cancer, thrombophilia and major trauma, and the presence of effective treatment might have had an impact on this outcome.

The significant strength of our investigation is a relatively large size of well-diagnosed individuals with IPF and cHP as well as the confirmation of VTE event by imaging studies. We are confident of the diagnosis of IPF and cHP in our cohort as all patients were re-evaluated in a multidisciplinary team discussion in terms of the currently published guidelines [14] or whether they would meet the criteria of cHP diagnosis according to the recently published reports [15, 17]. Moreover, we excluded from the study patients with known potential factors increasing the risk of VTE. Thus, the VTE incidence rate increased to 7.1/1000 person-years in cHP and to 11.8/1000 person-years in IPF compared to 1–2.7/1000 person-years in the general population [20–25], may indicate an association between these interstitial lung diseases and a prothrombotic state.

The aforementioned research papers concerning the link between VTE and ILD were medical registry-based studies [9–11, 13], with the exception for the study by Navaratnam et al. [12], where the diagnosis of IPF was made by clinicians who saw the patients. However, the diagnosis of VTE was established based on participants reports. Thus, potential limitation of these studies is misclassification of diagnoses as it was based on diagnostic code from the registry in the most of them.

There are some limitations of our study to consider. First and foremost, this was a retrospective, observational, single-center study and selection bias may have affected the outcomes of our research. Second, we reported only symptomatic VTE, as the investigations for VTE were based on symptoms and/or signs. We did not systematically screen for VTE all cases diagnosed with ILD. Additionally, VTE may have occurred in patients who were lost to follow-up. Thus, these factors might be the possible causes of underestimation of the incidence of VTE in patients with IPF and cHP. The authors are aware that the results of the study should be treated with caution due to statistical analyzes on a small group of patients due to the rarity of evaluated diseases. It is worth noting that symptomatic patients occurred with similar frequency in both cohorts, as the CT angiography was performed in comparable percentage of patients in both groups, and this reflects the real-world practice.

## Conclusions

In conclusion, our findings suggest that not only the patients with IPF, but also the patients with cHP have a marked increase in the risk of VTE. We believe that clinicians caring for patients with ILD should consider the occurrence of VTE in any IPF or cHP patient having the symptoms of worsening dyspnea, especially if no other cause can be identified.

Moreover, this raises the question if the other ILDs with a progressive fibrotic phenotype have an increased risk of VTE and how it should be screened. There is an urgent need for further prospective trials to examine the incidence and risk factors of VTE, the role of screening, anticoagulant prophylaxis, and management of patients with these disorders.

## Supplementary Information

**Additional file 1**. Detailed characteristics of patients with chronic hypersensitivity pneumonitis (Table S1) and patients with venous thromboembolism (Table S2).

## Data Availability

The datasets used and/or analysed during the current study are available from the corresponding author on reasonable request.

## Abbreviations

ASA: Acetylsalicylic acid
BALF: Bronchoalveolar lavage fluid
BMI: Body mass index
CI: Confidence interval
CT: Computed tomography
CTD-ILD: Connective tissue disease-associated interstitial lung disease
COPD: Chronic obstructive pulmonary disease
cHP: Chronic hypersensitivity pneumonitis
DLco: Diffusing capacity of the lung for carbon monoxide
DVT: Deep vein thrombosis
FVC: Forced vital capacity
FEV_1_: Forced expiratory volume in one second
GAP: Gender-age-physiology
GERD: Gastroesophageal reflux disease
HRCT: High resolution computed tomography
IIP: Idiopathic interstitial pneumonia
ILD: Interstitial lung disease
IPF: Idiopathic pulmonary fibrosis
LMWH: Low molecular weight heparin
NOAC: New oral anticoagulants
MDD: Multidisciplinary discussion
OR: Odds ratio
OSA: Obstructive sleep apnoea
PAH: Pulmonary arterial hypertension
PE: Pulmonary embolism
RR: Relative risk
SD: Standard deviation
SLB: Surgical lung biopsy
TBLB: Transbronchial lung biopsy
TLC: Total lung capacity
TVPG: Tricuspid valvular pressure gradient
UIP: Usual interstitial pneumonia
VTE: Venous thromboembolism
