# Supplementary material for: Chronic hypersensitivity pneumonitis is associated with an increased risk of venous thromboembolism: a retrospective cohort study
Source: BMC Pulm Med. 2021 Dec 17;21:416. doi: 10.1186/s12890-021-01794-y (PMC8684138; doi:10.1186/s12890-021-01794-y)
Supplement: Supplementary file 1 — Additional file 1. Detailed characteristics of patients with chronic hypersensitivity pneumonitis (Table S1) and patients with venous thromboembolism (Table S2). [file 12890_2021_1794_MOESM1_ESM.docx]

Additional file 1: Table S1. Detailed characteristics of patients with chronic hypersensitivity pneumonitis

| Characteristics | cHP patients n=152 |
| --- | --- |
|  |  |
| Age at diagnosis (y), mean (±SD) | 51.0 (±13.3) |
| Female, n (%) | 76 (50.0) |
| Ever smoker, n (%) | 49 (32.2) |
| Time from symptoms onset to diagnosis, mo, mean (±SD) | 36 (57,2) |
| Antigen exposure, n (%)  Poultry  Pigeons  Parrots  Hay/feed | 56 (37)  41 (27)  13 (9)  65 (43) |
| Precipitins present, n (%)  Farmer’s lung  Bird fancier’s lung | 19 (13)  33 (22) |
| HRCT  Fibrosis, any, n (%)  Reticular abnormalities +/- traction bronchiectasis, n (%)  Honeycombing, n (%)  No fibrosis n (%)  Not available initial assessment  BAL performed, n (%)  BALF cells’ count (M), mean (±SD)  BALF lymphocytes (%),mean (±SD)  BALF neutrophils (%),mean (±SD)  BALF eosinophils (%),mean (±SD)  BALF lymphocytosis >30%, n (%) | 86 (57)  62 (41)  24 (16)  57 (37)  9 (6)  121 (80)  36.13 (25,6)  45.8 (20.5)  6.6 (7.1)  2.7 (3.7)  98 (64) |
| TBLB performed, n (%) | 47 (31) |
| SLB performed, n (%)  Criobiopsy, n (%) | 49 (32)  9 (6) |

Abbreviations: BAL – bronchoalveolar lavage; BALF – bronchoalveolar lavage fluid; cHP – chronic hypersensitivity pneumonitis; HRCT – high resolution computed tomography; SLB – surgical lung biopsy; TBLB – transbronchial lung biopsy

Additional file 1: Table S2. Characteristics of patients with venous thromboembolism.

| **Patient**  **No**  **F/M** | **Age at VTE** | **Time from diagnosis of ILD to VTE (months)** | **FVC % pred** | **DLco % pred** | **D-dimer (ng/ml)** | **PASP**  **(Echo)** | **HRCT** | **Treatment of ILD at the time of VTE** | **Treatment of VTE** | **Bleeding episodes** |
| --- | --- | --- | --- | --- | --- | --- | --- | --- | --- | --- |
| **IPF** |  |  |  |  |  |  |  |  |  |  |
| **16 F** | 81 | 93 | NA | NA | 1628 | 61 | Probable UIP | No | LMWH | epistaxis |
| **52 M** | 77 | 25 | 53 | NA | 960 | 51 | UIP | No | LMWH/  NOAC | No |
| **130 F** | 60 | 0 | 83 | 25 | NA | 52 | UIP | No | LMWH/  NOAC | No |
| **147 F** | 77 | 0 | NA | NA | 13565 | 95 | UIP | No | LMWH/  NOAC | No |
| **175 M** | 64 | 22 | NA | NA | 4954 | 35 | UIP | No | LMWH/ Vitamin K antagonist | No |
| **210 M** | 53 | 3 | NA | NA | 1886 | 42 | UIP | P 40 mg/day AZA 150 mg/day | LMWH | No |
| **235 M** | 73 | 11 | NA | NA | NA | NA | UIP | Pirfenidone | LMWH | No |
| **260 M** | 74 | 0 | 78 | 47 | 551 | 31 | UIP | No | LMWH/Vitamin K antagonist | No |
| **288 M** | 78 | 27 | NA | NA | 1539 | 40 | UIP | P 40mg | No | No |
| **304 F** | 65 | 27 | NA | NA | 4855 | 36 | UIP | P 20 mg/day | LMWH | No |
| **319 F** | 75 | 108 | 64 | 24 | 7454 | 56 | UIP | No | LMWH/ NOAC | No |
| **324 M** | 73 | 73 | 89 | 35 | NA | 38 | UIP | No | Vitamin K antagonist | No |
| **cHP** |  |  |  |  |  |  |  |  |  |  |
| **36 F** | 55 | 37 | 53 | 43 | 182 | 41 | Fibrotic | P 10 mg/day | LMWH | No |
| **77 M** | 55 | 21 | 101 | 69 | 351 | 35 | Non-fibrotic | No | Vitamin K antagonist | No |
| **91 M** | 50 | 0 | 97 | 58 | NA | NA | Non-fibrotic | No | LMWH/ Vitamin K antagonist | No |
| **108 M** | 58 | 64 | 82 | 48 | NA | 35 | Fibrotic | Methylprednisolone 4 mg/day | LMWH | No |
| **175 M** | 63 | 37 | 94 | 72 | 2479 | 46 | Fibrotic | P 10 mg/day | LMWH/ NOAC | No |

Abbreviations: AZA – azathioprine; cHP – chronic hypersensitivity pneumonitis; DLCO – diffusing capacity of the lung for carbon monoxide; F – female, FVC – forced vital capacity; HRCT – high resolution computed tomography; IPF – idiopathic pulmonary fibrosis; LMWH – low molecular weight heparin; M- male; NOAC – new oral anticoagulant; P – prednisone, PASP - pulmonary arterial systolic pressure; VTE – venous thromboembolism; UIP – usual interstitial pneumonia;
